# Tackling Unwanted Proteolysis in Plant Production Hosts Used for Molecular Farming

**DOI:** 10.3389/fpls.2016.00267

**Published:** 2016-03-08

**Authors:** Manoj K. Mandal, Houtan Ahvari, Stefan Schillberg, Andreas Schiermeyer

**Affiliations:** Department of Plant Biotechnology, Fraunhofer Institute for Molecular Biology and Applied EcologyAachen, Germany

**Keywords:** antibodies, biopharmaceuticals, degradation, protease inhibitors, proteases, recombinant proteins, tobacco

## Abstract

Although the field of molecular farming has significantly matured over the last years, some obstacles still need to be resolved. A major limiting factor for a broader application of plant hosts for the production of valuable recombinant proteins is the low yield of intact recombinant proteins. These low yields are at least in part due to the action of endogenous plant proteases on the foreign recombinant proteins. This mini review will present the current knowledge of the proteolytic enzymes involved in the degradation of different target proteins and strategies that are applied to suppress undesirable proteolytic activities in order to safeguard recombinant proteins during the production process.

## Introduction

As the field of plant molecular farming has evolved over the last two decades, many obstacles have been overcome, leading to the first approval of a biopharmaceutical protein for human therapy in 2012 and additional candidates being evaluated in clinical trials (Paul and Ma, 2011). Although plant cells have been successfully engineered to humanize the N-glycan modification of recombinant proteins (Castilho and Steinkellner, 2012) and different protocols have been developed for cGMP compliant production (Fischer et al., 2012), an important issue limiting the broader adoption of plant molecular farming remains: the relatively low yield of recombinant proteins. Plant cells, especially the lytic vacuole and the apoplast, are rich in proteolytic enzymes of diverse classes (Goulet et al., 2012). Interestingly, the first approved biopharmaceutical protein made from plant cells is a lysosomal acidic beta-glucocerebrosidase, taliglucerase alfa, a human enzyme that is used in enzyme replacement therapy for Gaucher patients (Shaaltiel et al., 2007) and has evolved to withstand the harsh hydrolytic environment of the lysosome. Other target molecules like full-size IgG antibodies have frequently been reported to suffer from proteolytic degradation (Donini et al., 2015) irrespective of the plant system that has been used for production. In addition to reduced yields of the target protein, proteolytic processing might also lead to the formation of degradation products that have very similar physico-chemical properties as the intact target protein and are therefore difficult to remove during downstream processing. Several strategies are currently evaluated to safeguard the target protein against degradation that should ultimately lead to the development of improved plant host systems.

## Proteolytic degradation of recombinant proteins

Many candidate biopharmaceutical proteins such as plasminogen activators (Schiermeyer et al., 2005), cytokines (Sirko et al., 2011), human serum albumin (Sun et al., 2011), and monoclonal antibodies (Stevens et al., 2000; Sharp and Doran, 2001; Muynck et al., 2009) have been shown to undergo proteolytic processing to different degrees when they are produced in plant cells. The following section presents examples of the proteolytic degradation of recombinant proteins produced in plant cells and the proteolytic enzymes involved.

Monoclonal antibodies currently represent the largest class of biopharmaceuticals and thus also represent attractive target molecules for plant production platforms. However, plant-produced full-length antibodies often show degradation of their heavy chains, whereas the light chains usually remain intact. It has been known for a long time that plant cysteine proteases of the papain family are able to cleave immunoglobulins within the hinge region of their heavy chains to yield Fab and Fc fragments (Porter, 1959). Other proteases cleave immunoglobulins in the same region but at slightly different sites (Gorevic et al., 1985), which indicates that the cleavage depends not only on specific sequence recognition sites but also on the open and accessible conformation of the hinge region and, in some cases, other solvent-exposed loops. It is therefore not surprising that several research groups reported the processing of plant-produced recombinant IgG antibodies into Fc, Fab, F(ab')_2_ and other cleavage products (Hehle et al., 2014). Approximately 90% of the heavy chain of the human H10 IgG1λ monoclonal antibody was cleaved inside the cells of tobacco plants (Villani et al., 2009). The murine IgG1 monoclonal antibody MGR48 was cleaved in the hinge region under acidic conditions when it was spiked into crude leaf extracts from *Nicotiana tabacum* (Stevens et al., 2000). The authors of that study also noted that the proteolytic activity was higher in older leaves than in younger leaves. A systematic analysis of the murine antibody (IgG1) Guy's 13 produced in the tobacco production systems hairy roots, shooty teratoma, and suspension cells indicated that similar degradation products could be identified in all systems (Sharp and Doran, 2001). That study also established that the proteolytic processing occurs along the secretory pathway of the cell and in the apoplast. Similarly, degradation products of the chimeric human/rat IgG1κ LO-BM2 antibody heavy chain were identified in the intercellular wash fluid of transgenic *N. tabacum* plants and the spent cell culture medium of transgenic tobacco BY-2 suspension cells (Muynck et al., 2009). Most reports on the production of immunoglobulins in plants have been focused on the IgG1 isotype. However, for certain applications, other isotypes might also be of interest (Salfeld, 2007). A recent publication therefore compared the stability of human IgG1, IgG2, and IgG4 monoclonal antibodies in the spent culture medium of tobacco BY-2 suspension cells (Magy et al., 2014). This analysis revealed a significantly higher accumulation of the IgG1 isotype in the culture medium (10 mg/L) compared with the IgG2 (5.4 mg/L) and IgG4 (0.9 mg/L) isotypes. However, when the same set of antibodies was expressed in *Arabidopsis thaliana* suspension cells, no significant differences in accumulation were recognized. The accumulation of all isotypes was approximately 3 mg/L in the culture medium.

Because plant genomes encode several hundred proteolytic enzymes (van der Hoorn, 2008), it is challenging to identify the protease(s) that are responsible for the degradation of a given recombinant protein. It has been demonstrated that the proteolytic processing of the heavy chain of the human (IgG1κ) anti-HIV antibody 2F5 was effectively inhibited by phenylmethanesulfonyl fluoride (PMSF) or diisopropylfluorophosphate (DFP), two irreversible inhibitors of serine proteases (Mandal et al., 2014; Niemer et al., 2014). Similarly, it has been shown that the degradation of human IgG3 antibodies spiked into spent culture medium from tobacco BY-2 cells and other recombinant proteins, such as human α_1_-antitrypsin or BSA, spiked into the intercellular washing fluid of tobacco plants was partially inhibited by the addition of PMSF (Delannoy et al., 2008; Navarre et al., 2012; Castilho et al., 2014).

Because most pharmaceutical proteins are glycoproteins, their recombinant counterparts are targeted to the secretory route to obtain the desired glycan modification in the ER, Golgi apparatus and downstream compartments. Therefore, knowledge of secreted proteases and those residing in cell compartments along the secretory pathway is of critical importance to develop suitable strategies for the stabilization of recombinant proteins. Mass spectrometry based secretome analysis of tobacco BY-2 spent culture medium (Navarre et al., 2012), hydroponic culture medium of tobacco plants (Madeira et al., 2016; Wendlandt et al., 2016) and intercellular washing fluid of *N. benthamiana* leaves (Goulet et al., 2010a) revealed the presence of subtilisin-like proteases, serine carboxypeptidases, papain-like cysteine proteases (PLCP) and homologs of the CND41 aspartic protease belonging to the S8, S10, C1 and the A1 family of proteases according to the MEROPS classification (Rawlings et al., 2012). A proteomic survey of the spent culture medium from rice cells revealed the secretion of PLCPs, EP3A, and Rep-1 into the culture medium (Kim et al., 2008a). A specific member of the PLCP family, CysP6 from *N. tabacum*, has been implicated in the degradation of recombinant human interleukin-10 (IL-10) within the endoplasmic reticulum of the cell (Duwadi et al., 2015). A legumain-like cysteine protease belonging to the C13 family according to the MEROPS classification was most likely responsible for the degradation of recombinant equistatin produced in the leaf tissue of *Solanum tuberosum* (Outchkourov et al., 2003). The degradation of a recombinant plasminogen activator (DSPAα1) produced in tobacco cells has been shown to be reduced in the presence of EDTA, indicating the involvement of a matrix-metalloprotease in the degradation of DSPAα1 (Schiermeyer et al., 2005; Mandal et al., 2010). *In vitro* studies using recombinant proteolytic enzymes confirmed that two serine proteases, subtilisin (S8 family) and chymotrypsin (S1 family), and two PLCPs (C1 family), cathepsin B and cathepsin L, were able to cleave the 2F5 antibody HC within its CDR-H3 domain (Niemer et al., 2014).

## Strategies to combat proteolysis

During the past two decades various strategies have been developed and tested to reduce the proteolytic activity in a variety of plant expression systems to increase accumulation levels of recombinant biopharmaceuticals. The following sections describe these efforts in more detail and an overview of the different approaches to reduce the proteolytic activity in plant tissue and cell cultures is provided in Table 1.

**Table 1:** **Strategies to reduce proteolysis in plant tissues and cell cultures**.

		**Target protease(s)**	**Production host**	**Protein of interest**	**Effect on accumulation level**	**References**
**Supplementation of stabilizing agents**	Gelatin or polyvinylpyrrolidone		*N. tabacum* hairy roots	Murine IgG1 antibody	9-fold increase	Wongsamuth and Doran, 1997
	Polyvinylpyrrolidone		*N. tabacum* NT-1 cells	Murine IgG1 antibody	35-fold increase	LaCount et al., 1997
	BSA		*N. tabacum* NT-1 cells	hGM-CSF	2-fold increase	James et al., 2000
	HSA		*P. patens*	Human VEGF	3-fold increase	Baur et al., 2005
**Co-expression of protease inhibitors**	Cathepsin D inhibitor (*Sl*CDI)	Serine (S1) and aspartic proteases (A1)	*S. tuberosum*	α1-anti-chymotrypsin	2.5-fold increase	Goulet et al., 2010b
	*Sl*CDI or tomato cystatin SlCYS9	*Sl*CDI: serine (S1) and aspartic (A1) proteases. *Sl*CYS9: cysteine proteases (C1, C13)	*N. benthamiana*	C5-1 IgG monoclonal antibody	Up to 70–80% increase of antibody light chain	Goulet et al., 2012
	*Sl*CDI	Serine (S1) and aspartic proteases (A1)	*N. benthamiana*	C5-1 IgG monoclonal antibody	Up to 85% increase of antibody heavy chain	Goulet et al., 2012
	*Sl*CYS8	Cysteine proteases (C1)	*N. benthamiana*	C5-1 IgG monoclonal antibody	Up to 40% increase of mAb	Robert et al., 2013
	Oryzacystatin-I	Cysteine proteases (C1)	*N. tabacum*	Glutathione reductase (GR)	Increase of GR accumulation level not specified	Pillay et al., 2012
	Proteinase inhibitor II	Serine proteases (S1, S8)	*O. sativa* suspension cells	hGM-CSF	2-fold increase	Kim et al., 2008b
	Bowman-Birk serine protease inhibitor	Serine proteases (S1)	*N. tabacum* roots	Human single-chain IgG1 or full-size IgG4	2-2.5 fold increase	Komarnytsky et al., 2006
**Gene knockdown**	ihpRNA construct specific to Rep-1	Cysteine proteases (C1)	*O. sativa* suspension cells	hGM-CSF	2-fold increase	Kim et al., 2008a
	Expression of antisense sequences	Aspartic (A1), cysteine (C1), metallo- (M10) and serine proteases (S8)	*N. tabacum* BY-2 cells	IgG1κ antibody 2F5	4-fold increase of the intact antibody heavy chain	Mandal et al., 2014
	RNAi construct specific to CysP6	Cysteine protease (C1)	*N. tabacum*	Human IL-10	1.6-fold increase	Duwadi et al., 2015
**Fusion proteins**	Fusion of Zera domain		*N. tabacum* NT-1 cells	Zera domain fused subunit vaccine F1-V	3-fold increase	Alvarez et al., 2010
	Fusion of *Sl*CYS8		*N. benthamiana*	*Sl*CYS8 fused α1-ACT	25-fold increase	Sainsbury et al., 2013

## Supplementation of stabilizing agents

The use of suspension cultures for plant cells and organs to produce recombinant proteins enables the addition of protein-stabilizing agents to the culture medium. It has been demonstrated that the heavy chain of a murine IgG1 antibody could be stabilized in the spent culture medium of tobacco hairy roots by the addition of gelatin or polyvinylpyrrolidone (PVP), thereby increasing the production level up to nine-fold (Wongsamuth and Doran, 1997). Similarly the accumulation level of the anti-vitronectin human IgG1λ mAb M12 in the culture medium of hairy roots increased twofold when PVP was added to the culture medium (Häkkinen et al., 2014). The addition of PVP to the culture medium of transgenic tobacco NT-1 cells expressing a murine IgG1 antibody led even to a 35-fold increase in antibody heavy chain accumulation in the culture medium (LaCount et al., 1997). By supplementing the culture medium of transgenic tobacco NT-1 cells with bovine serum albumin, a two-fold increase in the accumulation of extracellular human GM-CSF was achieved (James et al., 2000). Likewise, the addition of human serum albumin to the culture medium of transgenic moss (*Physcomitrella patens*) cells expressing the human vascular endothelial growth factor enhanced its production levels three-fold (Baur et al., 2005). Whether the above-mentioned substances exert an inhibitory effect on proteolytic enzymes has not yet been analyzed, but the proteinaceous substances might act as an alternative substrate for extracellular proteases, thereby stabilizing the protein of interest.

## Co-expression of protease inhibitors

Different strategies have been tested to reduce the unwanted proteolysis of recombinant proteins in plant cells, such as the co-expression of protease inhibitors together with the protein of interest. In particular, protease inhibitors with specificity for cysteine, serine or aspartic proteases have been deployed for this purpose. It has been reported that co-expression of the Kunitz-type (I3 family according to the MEROPS classification) cathepsin D inhibitor (*Sl*CDI) from tomatoes with human α_1_-antichymotrypsin (α1-ACT) stabilizes the latter and leads to a 2.5-fold increase in its accumulation in potato leaves (Goulet et al., 2010b). In a follow-up study, the transient expression of *Sl*CDI or tomato cystatin *Sl*CYS9 (I25 family) was investigated for the potential to stabilize the murine C5-1 IgG monoclonal antibody in *N. benthamiana*. Whereas the expression of both inhibitors led to increased accumulation of the antibody light chain, higher production of the heavy chain could only be documented by the co-expression of *Sl*CDI (Goulet et al., 2012). In a recent report, another member of the tomato cystatin family, *Sl*CYS8, was used for transient co-expression with the C5-1 antibody in *N. benthamiana*. The accumulation of the C5-1 antibody increased approximately 40% on the whole plant scale. However, it has been recognized that the stabilizing effect of this cystatin is confined to the younger leaves of the plant. In older leaves, the *Sl*CYS8 levels were considerably lower and increased PLCPs activity has been documented in these leaves (Robert et al., 2013). Constitutive expression of the rice cysteine protease inhibitor oryzacystatin-I resulted in an increased accumulation and higher activity of the model protein glutathione reductase in tobacco plants compared with non-transgenic controls (Pillay et al., 2012). The co-expression of a synthetic construct containing trypsin and chymotrypsin inhibitor domains from the proteinase inhibitor II (I20 family) gene from *N. alata* with recombinant human granulocyte-macrophage colony-stimulating factor (hGM-CSF) led to a two-fold increase in the accumulation of secreted hGM-CSF in rice suspension cultures (Kim et al., 2008b). Co-secretion of the soybean Bowman-Birk serine protease inhibitor (I12 family) together with recombinant human single-chain IgG1 or full-size IgG4 antibodies from the roots of transgenic tobacco plants increased the accumulation of the antibodies 2- to 2.5-fold (Komarnytsky et al., 2006).

## Gene knockdown

Another promising strategy to reduce the proteolytic degradation of recombinant proteins is to knockdown the expression of protease-encoding genes. This strategy was introduced by Kim et al. (2008a) to suppress the expression of the cysteine protease gene Rep-1 by RNAi in rice suspension cells to improve the production of hGM-CSF. In that report, the authors used the rice amylase 3D promoter to drive the expression of hGM-CSF upon induction by sugar starvation. However, sugar starvation resulted in an accumulation of a cysteine protease from the C1A family encoded by the Rep-1 gene. The expression of Rep-1 was suppressed by post-transcriptional gene silencing using an intron-containing self-complementary hairpin RNA (ihpRNA) construct specific for Rep-1. This strategy resulted in a two-fold higher accumulation of hGM-CSF in the rice cell culture medium compared with its expression in a non-silenced cell line. A similar approach has been used for intact plants, where the RNAi-mediated silencing of another cysteine protease-encoding gene, CysP6, improved the accumulation of recombinant human IL-10 in tobacco leaves by approximately 1.6-fold (Duwadi et al., 2015). A somewhat broader protease silencing approach was followed by simultaneous silencing of four protease genes (NtAP, NtCP, NtMMP1, and NtSP) coding for proteases from four catalytic classes (aspartic, cysteine, metallo- and serine proteases) through the expression of the corresponding antisense sequences in tobacco BY-2 cells (Mandal et al., 2014). The study showed that the culture medium of the antisense RNA-expressing BY-2 cells had a lower level of total proteolytic activity than did wild-type BY-2 cells. When this transgenic BY-2 cell line was used to produce a recombinant full-length IgG1κ antibody, 2F5, it resulted in a four-fold higher accumulation of the intact antibody heavy chain compared with wild type cells expressing the same antibody.

With the introduction of different gene targeting strategies based on sequence-specific nucleases, it is now possible to disrupt any protease gene to completely knockout its activity (Fichtner et al., 2014). Although this technology has not yet been applied to protease genes, targeting has been used to knockout two α(1,3)-fucosyltransferases and two β(1,2)-xylosyltransferases in *N. benthamiana* to engineer plants that are devoid of plant-specific N-glycosylation patterns (Li et al., 2016). It is therefore only a matter of time before this technology will be applied to generate plants in which specific protease genes will be disrupted.

## Subcellular targeting and fusion proteins

To protect recombinant proteins from degradation in the apoplast or vacuole, targeting strategies have been developed to sequester the target protein from these hydrolytic cellular compartments. The retention of recombinant proteins in the ER or ER-derived structures has been proven to be particularly beneficial (Conrad and Fiedler, 1998). As plant seeds have evolved to store proteins in large quantities, a fusion strategy using the maize seed storage protein, γ-zein, has been developed. When the N-terminal Zera (γ-zein ER-accumulating) domain was fused to target proteins, the proteins accumulated in ER-derived protein bodies. The fusion of the Zera domain with the subunit vaccine F1-V from *Yersinia pestis* led to the formation of protein bodies and a three-fold higher accumulation of the fusion protein in tobacco NT-1 suspension cells compared to F1-V alone (Alvarez et al., 2010). Similar results were obtained in transiently transformed *N. benthamiana* and stably transformed alfalfa (*Medicago sativa*) plants. Likewise, the fusion of target proteins with hydrophobin I from *Trichoderma reesei* facilitated the formation of protein bodies in *N. tabacum* and *N. benthamiana* and increased the product yields (Joensuu et al., 2010; Gutiérrez et al., 2013).

In addition to the co-expression strategy with protease inhibitors described above, the tomato cystatin *Sl*CYS8 has been used to produce a fusion protein with human α1-ACT. This fusion protein accumulated at up to 25-fold higher levels compared with free α1-ACT (Sainsbury et al., 2013). However, in this case, the stabilizing effect was shown to be independent of the inhibition of cysteine proteases, as a fusion with a mutant, inactive, *Sl*CYS8 protein also displayed a similar stabilizing activity. The authors therefore speculated that the fusion with *Sl*CYS8 stabilizes the tertiary structure of α1-ACT and thereby prevents its attack by hydrolytic enzymes.

Based on the above-discussed strategies that can be used to prevent the proteolytic degradation of recombinant proteins, it is clear that there is no “magic bullet” that can stabilize all target proteins. Instead, an individual strategy has to be devised for each recombinant protein of interest. However, the tools described above should provide a suitable selection of procedures to tackle this important issue.

## Author contributions

All authors listed, have made substantial, direct and intellectual contribution to the work, and approved it for publication.

### Conflict of interest statement

The authors declare that the research was conducted in the absence of any commercial or financial relationships that could be construed as a potential conflict of interest.

## Abbreviations

ACT: antichymotrypsin
CDI: cathepsin D inhibitor
DFP: diisopropylfluorophosphate
DSPA: *Desmodus rotundus* plasminogen activator
ERT: enzyme replacement therapy
Fab: fragment antigen-binding
Fc: fragment crystallizable
GM-CSF: granulocyte-macrophage colony-stimulating factor
HC: heavy chain
Ig: immunoglobuline
IL-10: interleukin-10
mAb: monoclonal antibody
PLCP: papain-like cysteine protease
PMSF: phenylmethanesulfonyl fluoride
PVP: polyvinylpyrrolidone.
